# Development and validation of women’s environmental health scales in Korea: severity, susceptibility, response efficacy, self-efficacy, benefit, barrier, personal health behavior, and community health behavior scales

**DOI:** 10.4069/kjwhn.2021.06.21

**Published:** 2021-06-30

**Authors:** Hee Kyung Kim, Hyun Kyoung Kim

**Affiliations:** Department of Nursing, Kongju National University, Gongju, Korea

**Keywords:** Environmental health, Health behavior, Psychometrics, Women’s health

## Abstract

**Purpose:**

This study aimed to develop the following scales on women’s environmental health and to examine their validity and reliability: severity, susceptibility, response efficacy, self-efficacy, benefit, barrier, personal health behavior, and community health behavior scales.

**Methods:**

The item pool was generated based on related scales, a wide literature review, and in-depth interviews on women’s environmental health according to the revised Rogers’ protection motivation theory model. Content validity was verified by three nursing professionals. Exploratory factor analysis, convergent validity, and internal consistency reliability were examined.

**Results:**

The scales included 10 items on severity, 11 on susceptibility, 10 on response efficacy, 14 on self-efficacy, 8 on benefits, 10 on barriers, 17 on personal health behavior, and 16 on community health behavior. Convergent validity with the environmental behavior scale for female adolescents was supported. The Cronbach’s α values for internal consistency were good for all scales: severity, .84; susceptibility, .92; response efficacy, .88; self-efficacy, .90; benefits, .91; barriers, .85; personal health behavior, .90; and community health behavior, .91.

**Conclusion:**

The evaluation of the psychometric properties shows that these scales are valid and reliable measures of women’s environmental health awareness and behaviors. These scales may be helpful for assessing women’s environmental health behaviors, thereby contributing to efforts to promote environmental health.

## Introduction

Although evidence is accumulating that women’s environmental health problems are caused by environmental pollution [1-5], few studies have investigated health behaviors that promote women’s environmental health [6]. Liu et al. [5] measured levels of exposure to environmental pollutants but did not address lifestyle changes, which is a limitation of that study. Therefore, it is necessary to investigate various aspects of health behavior. Women’s environmental health problems may affect the reproductive organs from birth to old age, reflecting the need to protect women’s health in advance from contaminants to which women are repeatedly exposed during the course of their life [7]. A useful tool to measure environmental health behavior is required to inform efforts to improve women’s reproductive health. However, the existing tools for measuring women’s environmental health behavior have limitations, such as being restricted to specific behaviors (i.e., not including awareness) [8], having adolescent participants [9], and dealing with unrelated health behaviors [10].

**Figure 1.**
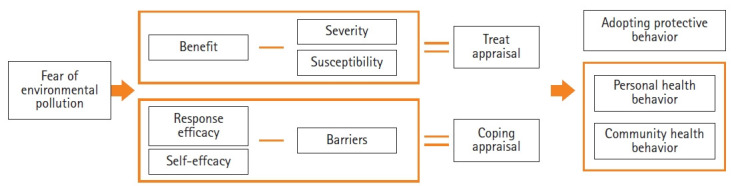
Conceptual framework of environmental health awareness and behavior scales for women.

This study developed a tool based on Rogers’ [11] revised protection motivation theory to explain the mechanism of environmental health awareness, which affects environmental health behavior. When humans have fears regarding environmental health, they adopt protective behavior through threat appraisal and coping appraisal. Threat appraisal involves subtracting perceived severity and perceived vulnerability from the rewards of maladaptive responses, and coping appraisal involves subtracting the costs of adaptive responses from response efficacy and self-efficacy [11]. Severity is defined as one’s evaluation of fear of a severe negative outcome, vulnerability as perceptions regarding the mortality or morbidity of the disease, response efficacy as the effect that behavior would have on disease prevention, and self-efficacy as an evaluation of the individual’s ability to engage in certain behavior [12]. Rogers [11] extended this theoretical framework to emphasize the rewards of maladaptive responses and adaptive responses. The rewards of maladaptive responses are defined as the benefits of continuing a risky behavior and the costs of adaptive responses as the losses induced by maintaining a protective behavior [12]. Fear of environmental risks stimulates women’s awareness of the severity of environmental harm, their susceptibility to environmental diseases, the response efficacy of preventive behavior, their self-efficacy, the rewards of continuing environmental behavior, and the costs of maladaptive behaviors that are dismissive of environmental health. Rogers’ revised theory has been verified in various fields such as chronic pediatric diseases [13] and sexually transmitted infections [14]. Rogers’ theory can also be adopted in environmental health-related fields because it provides insights into the inner decision-making mechanism for coping with threats [12]. Therefore, the revised protection motivation theory was applied in this study to measure women’s internal perceptions and actions regarding environmental threats (Figure 1).

This study aimed to develop eight scales to measure environmental health awareness and health behavior (severity, susceptibility, response efficacy, self-efficacy, benefits, barriers, personal health behavior, and community health behavior) by applying the method developed by DeVellis [15] for women residing in local communities according to Rogers’ revised protection motivation theory [11]. It is hoped that these scales will be used to measure the effectiveness of interventions for women’s environmental health awareness and health behavior. The specific purposes were as follows: (1) to develop severity, susceptibility, response efficacy, self-efficacy, benefit, barrier, personal health behavior, and community health behavior scales for women’s environmental health; and (2) to confirm the validity and reliability of the measurement tools.

## Methods

Ethics statement: This study was reviewed by the Institutional Review Board of Kongju National University (KNU-IRB-2020-34) and adhered to the Declaration of Helsinki. Informed consent was obtained from participants.

### Study design

This is a methodological study to develop and validate the following eight scales for women’s environmental health in Korea: severity, susceptibility, response efficacy, self-efficacy, benefit, barrier, personal health behavior, and community health behavior scales.

### Participants

The inclusion criteria were Korean women over the age of 19 years who lived in local communities. The criteria for selection were women who could speak, write, and read Korean and those who agreed with the purpose and process of the study. The exclusion criteria were women currently hospitalized for health problems, and those who had difficulty understanding the purpose and content of the study.

### Development of the preliminary items

To develop the preliminary items, existing tools, a related literature review, and interview data from 10 women in the local community were analyzed. The literature review was done from September 9 to 13, 2020 using PubMed, CINAHL, Education Resources Information Center (ERIC), and the Research Information Sharing Service of Korea. For each database, an advanced search —(“Environment”[Mesh] AND (“Health Behavior”[Mesh] AND (“ Wom*”[Mesh] OR “Female”[Mesh]) AND “Psychometrics”[Mesh]),” “Environmental health”[Mesh] AND “severity” AND (“Women”[Mesh] OR “Female”[Mesh]) AND “Psychometrics”[Mesh],” “Environmental health”[Mesh] AND “vulnerability OR susceptibility” AND (“Women”[Mesh] OR “Female”[Mesh]) AND “Psychometrics”[Mesh],” “Environmental health”[Mesh] AND “self-efficacy” AND (“Women”[Mesh] OR “Female”[Mesh]) AND “Psychometrics”[Mesh],” “Environmental health”[Mesh] AND “self-efficacy” AND (“Women”[Mesh] OR “Female”[Mesh]) AND “Psychometrics”[Mesh],” “Environmental health”[Mesh] AND “benefit OR reward” AND (“Women”[Mesh] OR “Female”[Mesh]) AND “Psychometrics”[Mesh],” “Environmental health”[Mesh] AND “cost OR barrier” AND (“Women”[Mesh] OR “Female”[Mesh]) AND “Psychometrics”[Mesh],” “Environmental health”[Mesh] AND “behavior” AND (“Women”[Mesh] OR “Female”[Mesh]) AND “Psychometrics”[Mesh]”—and a keyword search for (‘Environment,’ ‘Health Behavior,’ ‘Female,’ ‘Measurement,’ and ‘Tools’) were done. The search of the four databases yielded 27, 10, one, and three results, respectively. Four articles were also retrieved through a manual search. Finally, three tools were used [8-10]. The interviews were held from September 26 to October 29, 2020. The researcher interviewed two women face-to-face and eight women by phone. The interviews took an average of 40 minutes per person, and two interviews were conducted for each participant. Participants were recruited through convenience sampling, and the face-to-face interviews were conducted at the office of the health center. The women’s age ranged from 23 to 43 years, with an average of 37.4 years. The interviewees comprised four housekeepers, two freelancers, one bank clerk, one researcher, and two educators. Nine of the women had no health problems, while one had diabetes. The questionnaire was guided through a semi-structured questionnaire, with prompts such as “Please tell me about environmental pollutants that pose a threat to health.” The final 106 meaningful statements were derived, listed, and allocated to the scales. In total, 101 items of the preceding tools [8-10] were modified according to Rogers’ revised protection motivation theory [16]. The conceptual framework of the tool was modified into eight scales: severity, susceptibility (adapted from vulnerability), response efficacy, self-efficacy, benefits (adapted from rewards of maladaptive responses), barriers (adapted from costs of adaptive responses), personal health behavior, and community health behavior (Figure 1). A Likert scale was used, with responses from 1 (not at all) to 5 (very much). In the item extraction process, the researchers independently extracted and then decided whether to include inconsistent items through a meeting. Items with disagreements were included in the request for expert review of content validity. Fifty overlapping items were removed from the interview data, and the final 157 preliminary items were developed.

The content validity of the preliminary items was verified by one head of a women’s hospital, one professor of women’s health nursing, and one maternal and child health expert at a public health center. Their average age was 52.2 years, and their average professional experience was 26.7 years. A request was made via e-mail for them to review content validity using a 5-point Likert scale from 1 (not very valid) to 5 (very valid). The item-level content validity index (I-CVI) and the average scale-level content validity index (S-CVI/Ave) were tested. For the I-CVI, the ratio of ‘valid’ and ‘very valid’ for each item was set as .78 or more, and for the S-CVI/Ave, the average of I-CVI for the item was set as .90 or more [16].

### Preliminary survey

The preliminary survey was done from November 19 to 23, 2020. Two women who met the inclusion criteria (22 years old and 61 years old) read the questions one by one to verify whether they understood the meaning and to confirm their understanding. The degree of comprehension was evaluated from 1 point (‘I do not understand at all’) to 5 points (‘I understand very well’). The average score of comprehension was 4.5 points, and no item was rated as difficult to understand. The average time required to respond was 15 minutes.

### Measurement tools for convergent validity

The 43-item Environmental Health Perception for Female Adolescents (EHP-FA) tool, developed to evaluate the environmental health awareness of female adolescents aged 18 to 22 years [9], was used for convergent validity. This tool is comprised of four subscales: sensitivity (17 items), vulnerability (8), response efficacy (9), and self-efficacy (9) according to Rogers’ original theory [17]. At the time of development, Cronbach’s α was .94, .95, .88, and .90 for each subscale; and in this study, the corresponding values were .85, .76, .87, and .86, respectively. The 32-item Environmental Health Behavior for Female Adolescents (EHB-FA) [9] tool was also used. The EHP-BA was developed to evaluate the environmental health behaviors of female adolescents aged 18 to 22 years and has two subscales: personal health behavior (19 items) and community health behavior (13). Permission for use was obtained. At the time of development, Cronbach’s α was .94 and .88 for each subscale, respectively; and in this study, it was .92 and .89, respectively. The intention-related measurement used for the validity test was rated on a 7-point Likert scale, with responses ranging from 1 point (‘I am not familiar with environmental health behavior’) to 7 points (‘I regularly practice environmental health behavior’) [8].

### Data collection

From November 27 to December 3, 2020, survey data were collected by two researchers and three research assistants at schools, welfare centers, academies, libraries, public health centers, and homes in Daejeon, Gongju, and Sejong in Korea. The research assistants met with potential participants, explained the research purpose, and received signed informed consent forms.

According to the sample size of 200 to 400 persons proposed in exploratory factor analysis to verify construct validity [18], the required number of participants was 200. Considering a possible drop-out rate of 10%, the questionnaire was distributed to 220 women. After excluding 10 inappropriate responses, data from 210 (95.5%) women were analyzed.

### Data analysis

The collected data were analyzed using SPSS ver. 25.0 (IBM Corp., Armonk, NY, USA). Exploratory factor analysis and promax rotation in principal axis factor analysis were used due to the correlations between factors. To confirm the appropriateness of the sample, the Kaiser-Mayer-Olkin (KMO) and Bartlett sphericity tests were performed. The criterion for item selection for factor extraction was that the eigenvalue was greater than 1 and the commonality of each item was .40 or more [19]. Subscale intercorrelations and the item total correlation (ITC) were examined [20]. Pearson’s correlation coefficients with the EHP-FA and EHB-FA were used for convergent validity analysis. A Cronbach’s α of .70 or higher was considered to indicate reliability [21].

## Results

### Demographic characteristics of the participants

The average age of the participants was 36.14 (standard deviation, ±13.76) years (range, 19–70 years), and 54.3% of the participants had a spouse. The proportion of high school graduates was 46.7%, and 51.0% did not have a job. The most common range of monthly household income was between 4.5 million Korean won (approximately 4,000 US dollars) and 6 million Korean won (5,300 US dollars), which accounted for 30.5% of the participants. Some of the participants had previous experiences of disease treatment (35.7%), and 22.9% of the participants had a disease at the time of the survey (Table 1).

### Content validity

The content validity of each item was .80–1.00 for 152 of the 157 items, and five items had an I-CVI less than .78. As the researchers reviewed the items, the five items with an I-CVI content validity less than .78 based on the expert group review were deleted. The deleted items were ‘Ask for health information,’ ‘I have a habit of exercising,’ ‘I decide on my own health behavior,’ ‘Move to a place with less pollution,’ and ‘Endometriosis may occur.’ The S-CVI/Ave of the final 152 questions was .92. The researchers held a meeting to ensure that the final items conveyed the intended meaning. The final 152 preliminary items included 24 on severity, 12 on susceptibility, 13 on response efficacy, 14 on self-efficacy, 10 on benefits, 18 on barriers, 33 on personal behavior, and 18 on community behavior.

### Factor analysis

Exploratory factor analysis was performed by applying principal axis factor analysis and promax rotation for each of the eight conceptual scales of the 152 preliminary items selected according to their content validity.

#### Severity of environmental health risks

The KMO value was .83, exceeding the standard value of .80, and Bartlett’s sphericity test showed that the approximate chi-square value was 743.82 (degree of freedom [df]=45, *p*<.001). After factor analysis, items with the following values were extracted: an eigenvalue of 1 or more, a commonality of .40 or more, a subscale intercorrelation coefficient between factors of .30–.80, an ITC of .40 or more, and items that met the criteria, in which one factor includes three or more items. Four items were eliminated on the severity scale. As a result, this subscale consisted of four items for the first factor (‘chemicals’), three items for the second factor (‘electromagnetic waves’), and three items for the third factor (‘harmful food’). The correlations between all three factors ranged from .43 to .48 (*p*<.001) and the correlations between the scale and subscales were .88, .79, and .76, respectively (*p*<.001). The ITCs ranged from .58 to .74 (*p*<.001), and the explained variance was 65.4% (Table 2, Supplementary Table 1).

#### Susceptibility to environmental health problems

The KMO value of the susceptibility scale was .87, and Bartlett’s chi-square value was 991.91 (df=55, *p*<.001); these values were suitable. After factor analysis of 12 items, seven items were included in the first factor (‘reproductive health problems’) and three items for the second factor (‘general health problems’). One item (‘ovarian problems’) was eliminated due to low commonality. The correlation between the final factors was .60 (*p*<.001), and the correlations between the scale and subscales were .95 and .86, respectively (*p*<.001). The ITCs ranged from .56 to .85 (*p*<.001), and the explained variance was 69.8% (Table 2, Supplementary Table 1).

#### Response efficacy related to environmental health behaviors

Suitable values were found for the KMO test (.88) and Bartlett’s chi-square value (896.72; df=45, *p*<.001). After factor analysis of 13 items, seven items for the first factor (‘avoiding toxicants’), and three items for the second factor (‘pursuit of health’) were selected. Three items related to ‘vegetable consumption,’ ‘migrating to a low-pollution area,’ and ‘inquiry to medical staff’ were eliminated because they had ITCs of less than .40. The correlation between the final factors was .59 (*p*<.001), and the correlations between the scale and subscales were .97 and .86, respectively (*p*<.001). The ITCs ranged from .50 to .73 (*p*<.001), and the explained variance was 60.3% (Table 2, Supplementary Table 1).

#### Self-efficacy related to environmental health behaviors

The KMO value was .87, and Bartlett’s chi-square value was found to be 874.90 (df=91, *p*<.001). After the factor analysis of 14 questions, all questions were selected. There were five items for the first factor (‘preventive efficacy’), five items for the second factor (‘judgment efficacy’), and four items for the third factor (‘control efficacy’). The correlations between the factors ranged from .40 to .48 (*p*<.001), and the correlations between the scale and subscales were .84, .86, and .73, respectively (*p*<.001). The ITCs ranged from .48 to .72 (*p*<.001), and the explained variance was 67.2% (Table 2, Supplementary Table 1).

#### Benefits of environmental health behaviors

This scale was found to be suitable, with a KMO value of .88 and a Bartlett’s chi-square value of 1,074.58 (df=595, *p*<.001). After the factor analysis of 10 items, eight items were selected: five items for the first factor (‘psychological benefits’), and three items for the second factor (‘physical benefits’). The correlation between the final factors was .50 (*p*<.001), and the correlations between the scale and subscales were .82 and .92, respectively (*p*<.001). The ITCs ranged from .47 to .80 (*p*<.001), and the explained variance was 75.8% (Table 2, Supplementary Table 1).

#### Barriers to environmental health behaviors

The scale on barriers was found to be suitable, with a KMO value of .85 and Bartlett’s chi-square value of 764.68 (df=45, *p*<.001). After the factor analysis of 18 items, five items for the first factor (‘negative atmosphere’) and five items for the second factor (‘burden’) were selected. The correlation between the factors was .45 (*p*<.001), and the correlations between the scale and subscales were .87 and .84, respectively (*p*<.001). The ITCs ranged from .40 to .70 (*p*<.001), and the explained variance was 58.5% (Table 2, Supplementary Table 1).

#### Personal health behavior

Suitable results were found for the KMO value (.88) and Bartlett’s chi-square value (2154.69; df=153, *p*<.001). After the factor analysis of 33 items, 17 items were selected: seven items for the first factor (‘lifestyle’), four items for the second factor (‘personal goods’), three items for the third factor (‘food’), and three items for the fourth factor (‘dust’). The correlations between the factors ranged from .40 to .48 (*p*<.001), and the correlations between the scale and subscales were .87, .77, .82, and .61, respectively (*p*<.001). The ITCs ranged from .48 to .76 (*p*<.001), and the explained variance was 67.7% [20] (Table 2, Supplementary Table 1).

#### Community health behavior

For the community health behavior scale, the KMO value was .87 and Bartlett’s chi-square value was 1,788.85 (df=120, *p*<.001). After the factor analysis of 18 items, 16 items were selected: five items for the first factor (‘reduction’), five items for the second factor (‘involvement’), three items for the third factor (‘recycling’), and three items for the fourth factor (‘reuse’). The correlations between the factors were .40 to .55 (*p*<.001) and the correlations between the scale and subscales were .85, .84, .68, and .77, respectively (*p*<.001). The ITCs ranged from .55 to .77 (*p*<.001), and the explained variance was 68.8% (Table 2, Supplementary Table 1).

The finally developed eight scales included 10 items on severity, 11 on susceptibility, 10 on response efficacy, 14 on self-efficacy, 8 on benefits, 10 on barriers, 17 on personal health behavior, and 16 on community health behavior (Table 2).

### Reliability

Cronbach’s α (95% confidence interval [CI]), as a measure of internal consistency, was good for all scales and subscales (Supplementary Table 1):

· Severity: .84 (95% CI=.82–.95; chemicals=.80; electromagnetic waves=.74; harmful food=.70)
· Susceptibility: .92 (95% CI=.90–.94; reproductive health problems=.94; general health problems=.78)
· Response efficacy: .88 (95% CI=.86–.91; avoiding toxicants=.87; pursuit of health=.76)
· Self-efficacy: .90 (95% CI=.88–.91; preventive efficacy=.88; judgment efficacy=.86; control efficacy=.80)
· Benefits: .91 (95% CI=.89–.93; psychological benefits=.91; physical benefits=.85)
· Barriers: .85 (95% CI=.82–.88; negative atmosphere=.83; burden=.81)
· Personal health behavior: .90 (95% CI=.88–.92; lifestyle=.90; personal goods=.84; food=.82; dust=.81)
· Community health behavior: .91 (95% CI=.89–.93; reduction=.89; involvement=.83; recycling=.77; reuse=.77)

### Convergent validity

The developed tool showed statistically significant positive correlations with EHP-FA and EHB-FA, demonstrating convergent validity. All of the scales showed a significant positive correlation with the EHP-FA: severity (r=.62, *p*<.001), susceptibility (r=.65, *p*<.001), response efficacy (r=.78, *p*<.001), self-efficacy (r=.66, *p*<.001), benefits (r=.29, *p*<.001), barriers (r=.21, *p*=.002), personal behavior (r=.40, *p*<.001), and community behavior for women’s environmental health (r=.39, *p*<.001). A significant positive correlation with the EHB-FA was also found: severity (r=.23, *p*=.001), susceptibility (r=.55, *p*<.001), response efficacy (r=.26, *p*<.001), self-efficacy (r=.51, *p*<.001), benefits (r=.18, *p*=.001), barriers (r=.10, *p*=.013), personal behavior (r=.92, *p*<.001), and community behavior for women’s environmental health (r=.90, *p*<.001) (Table 3).

## Discussion

The scales developed in this study were based on the revised protection motivation theory [11]. This theory explains changes in health behavior by adding the concepts of self-efficacy, rewards of maladaptive responses, and costs of adaptive responses to its original theoretical form [17]. When a person feels that the action’s reward exceeds its cost based on threat appraisal and coping appraisal, he or she can intend to take action and change the behavior [11]. In this study, all concepts of the revised protection motivation theory were substituted with corresponding environmental health concepts, and psychological evidence for the composition of the tool was confirmed [15].

As a result of reviewing existing environmental health behavior measurement tools [11,12,15], a literature review, interviews, and expert content validity test, it was found that the I-CVI and S-CVI/Ave values were above the corresponding standards, thereby establishing content validity [16]. Construct validity was confirmed through empirical tests of the pattern matrix, the structural matrix, the correlation coefficients between all items and each factor, and the correlation coefficients between factors [19].

This study attempted to grasp the meaning of the factors for the concepts underlying each scale. The severity scale comprised three subscales (‘chemicals,’ ‘electromagnetic waves,’ and ‘harmful food’). A difference between this scale and existing tools is that the severity of electromagnetic waves was derived as one factor. The items related to microplastics and light pollution reflect the recent problem of environmental pollution. Severity is an important concept related to environmental health in the United States, as a previous study assessed whether people had been exposed to environmental toxicity in the clinic [22]. Furthermore, this tool can be easily used as a more straightforward question.

The susceptibility scale included two subscales (‘reproductive health problems’ and ‘general health problems’). It can be seen that women were aware of their reproductive health problems and the health problems of the fetus and their children. As the relationship between female reproductive health problems and environmental pollutants has recently been established [2,23], it can be seen that this scale reflects the environmental health perceptions of women residing in local communities.

Response efficacy contained two subscales (‘avoiding toxicants’ and ‘pursuit of health’). The fact that the actions to avoid environmental pollutants had a higher explanatory power than actions taken to pursue health is consistent with the principle of precautionary care used in environmental health [7,24].

Self-efficacy contained three subscales (‘preventive efficacy,’ ‘judgment efficacy,’ and ‘control efficacy’). This classification does not exist in existing tools. Since self-efficacy is a strongly influential variable within the theory of health behavior changes [25], the developed scale will be valuable as a measurement tool.

Benefits consisted of two subscales (‘psychological benefits’ and ‘physical benefits’). It semantically coincided with the concept of compensation for actions, which has recently been proposed in the revised motivational theory [11]. In addition, the concept of benefits includes the possibility of measuring rewards for actions that existing tools cannot measure.

Barriers had two subscales (‘negative atmosphere’ and ‘burden’). The classification of items was appropriate in terms of content and semantically coincided with the concept of the cost of action, which has recently been proposed in the revised motivational theory [11]. According to the theory of change in health behavior, people are more sensitive to the barriers of health behavior than to the benefits of behavior [12]. Therefore, the scale dealing with barriers should be included in studies using the measurement tool developed in this study.

Personal health behavior consisted of four subscales (‘lifestyle,’ ‘personal goods,’ ‘food,’ and ‘dust’). Compared to other tools [8], the personal behavior scale contains health behaviors that are easy for women to practice in daily life; therefore, it is straightforward to measure health behaviors using this tool, which is advantageous.

Community health behavior consisted of four subscales (‘reduction,’ ‘involvement,’ ‘recycling,’ and ‘reuse’). This subscale reflects the community’s commitment to creating an environment that is not harmful to health by preventing environmental pollution [26].

The measurement tool developed in this study utilized all the constituent factors of the revised protection motivation theory model. Furthermore, validity and reliability testing was done. The tool reflects a comprehensive array of information on the environmental awareness and behavior of women residing in local communities in Korea through interviews and surveys. Therefore, it is distinctive from existing tools. It can be used to measure women’s environmental health awareness and strengthen environmental health behavior.

A limitation of this study limitation is the difficulty of generalizing the results to women in Korea as a whole or in other countries because data were collected from a local community setting in Korea. The convergent validity may have been high because of the lack of a gold-standard tool and because it was developed by applying a revised theory based on existing tools. Additionally, confirmatory factor analysis was not conducted.

We suggest conducting confirmatory factor analysis in further research to test the fitness of the theoretical framework. In the exploratory factor analysis, the variance explained by each subscale was found to be from 58.5% to 75.8%. Further efforts to find the remaining sources of variance are needed. Additional analyses of women from a wider variety of regions are required to address the limitation of generalizability. It is also necessary to investigate whether women with environmental health problems have high scores on these scales.

This study developed the following scales for measuring women’s environmental health: severity, susceptibility, response efficacy, self-efficacy, benefit, barrier, personal behavior, and community behavior for women residing in local communities in Korea. Research on environmental health in women has attracted increasing attention not only in Korea but also worldwide. As the scales’ validity and reliability were verified from various angles, their suitability for use in future research on women’s environmental health can be confirmed.

## Figures and Tables

**Table 1.** Demographic characteristics of the participants (N=210)

Variable	Categories	n (%)	Mean±SD
Age (year)	19	4 (1.9)	36.14±13.76
	20–29	81 (38.6)	
	30–39	39 (18.6)	
	40–49	52 (24.8)	
	50–59	21 (10.0)	
	≥60	13 (6.1)	
Spouse	Yes	114 (54.3)	
	No	96 (45.7)	
Education	Elementary school	2 (1.0)	
	Middle school	4 (1.9)	
	High school	98 (46.7)	
	Associate or bachelor’s	79 (37.6)	
	Master’s or doctoral	27 (12.9)	
Job	Yes	103 (49.0)	
	No	107 (51.0)	
Monthly household income (KRW)	≤1.5 million	31 (14.8)	
	1.5–3 million	39 (18.6)	
	3.01–4.5 million	39 (18.6)	
	4.51–6 million	64 (30.5)	
	≥6.01 million	37 (17.6)	
Medical history^†^	Yes	75 (35.7)	
	No	135 (64.3)	
Present disease^‡^	Yes	48 (22.9)	
	No	162 (77.1)	

KRW: Korean won (1 million = KRW is approximately 900 US dollars).

† Immune disease (11), uterine myoma (10), cancer (8), arthritis (7), indigestion (7), ovarian disease (6), pneumonia (4), thyroid disease (3), others (19);

‡ Arthritis (9), hypertension (7), immune disease (6), thyroid disease (6), ovarian disease (4), others (16).

**Table 2.** Exploratory factor analysis of severity, susceptibility, response efficacy, self-efficacy, benefit, barrier, personal behavior, and community behavior for women’s environmental health (N=210)

Scales	Items	Factor 1	Factor 2	Factor 3
		Chemicals	Electromagnetic waves	Harmful food
Severity	12. Nail polish	.87	.28	.35
	9. Plastic products	.83	.45	.40
	10. New furniture	.81	.41	.41
	11. Perfume	.62	.58	.28
	8. Night lighting	.44	.86	.38
	7. Cell phone	.23	.77	.25
	23. Microwave heating	.55	.75	.51
	18. Microplastics	.35	.30	.84
	19. Food additives	.41	.31	.78
	22. Instant food	.30	.41	.73
Eigenvalue		4.26	1.17	1.11
Total variance explained=65.4%				
Kaiser-Meyer-Olkin=.83, Bartlett’s test of sphericity=743.82, degree s of freedom=45, *p*<.001				

Scales	Items	Factor 1	Factor 2
		Reproductive health problems	General health problems
Susceptibility	3. Gynecologic cancer	.91	.46
	2. Infertility	.90	.55
	4. Menstrual problems	.88	.45
	1. Uterine disease	.87	.52
	6. Fetal anomaly	.80	.54
	7. Abortion	.79	.57
	8. Precocious puberty	.75	.54
	9. Atopic dermatitis	.55	.83
	10. Respiratory disease	.30	.76
	12. Immunosupression	.65	.75
	11. Obesity	.62	.74
Eigenvalue		6.53	.1.15
Total variance explained=69.8%			
Kaiser-Meyer-Olkin=.87, Bartlett’s test of sphericity=991.91, degrees of freedom=55, *p*<.001			

Scales	Items	Factor 1	Factor 2
		Avoid toxicant	Pursuit of health
Response efficacy	1. Avoid electromagnetic waves	.81	.38
	12. Avoid micro-dust	.80	.40
	6. Avoid chemicals	.79	.41
	7. Avoid instant food	.70	.63
	8. Avoid night lights	.69	.58
	11. Home cooking	.68	.66
	5. Personal hygiene	.66	.55
	2. Attention to environmental issues	.45	.83
	3. Environmental information seeking	.38	.80
	4. Try to drink clean water	.48	.77
Eigenvalue		4.93	1.10
Total variance explained=60.3%			
Kaiser-Meyer-Olkin=.88, Bartlett’s test of sphericity=896.72, degrees of freedom=45, *p*<.001			

Scales	Items (I can prevent...)	Factor 1	Factor 2	Factor 3
		Preventive efficacy	Judgement efficacy	Control efficacy
Self-efficacy	9. toxicants via the air	.89	.42	.30
	6. toxicants through my skin	.84	.38	.09
	7. toxicants via soil	.82	.47	.37
	8. toxicants via food	.81	.43	.46
	5 hazardous environment	.70	.35	.64
	10. I scrutinize consumer information.	.38	.85	.40
	11. I search for internet information.	.37	.82	.44
	14. I can judge environmental news.	.32	.80	.55
	12. I learn about environmental toxicant.	.55	.78	.29
	13. I look for food hazards.	.52	.76	.37
	3. I choose useful health behavior	.22	.44	.82
	2. I manage my health	.26	.42	.81
	1. I identify harmful environment	.44	.43	.76
	4. I avoid temptations of convenience	.24	.28	.70
Eigenvalue		6.21	1.85	1.34
Total variance explained=67.2%				
Kaiser-Meyer-Olkin=.87, Bartlett’s test of sphericity=874.90, degrees of freedom=91, *p*<.001				

Scales	Items	Factor 1	Factor 2
		Psychological benefits	Physical benefits
Benefits	10. I feel comforted that environmental health is beneficial to my health.	.91	.38
	9. I feel satisfied that I did not harm the environment.	.90	.39
	8. I feel psychological stability about the environment.	.84	.47
	7. The guilt of environmental pollution is relieved.	.84	.41
	6. Environmental protection is a social phenomenon.	.78	.54
	5. Digestion functions well.	.46	.90
	3. Weight is lost.	.33	.88
	4. Skin improves.	.57	.84
Eigenvalue		4.70	1.36
Total variance explained=75.8%			
Kaiser-Meyer-Olkin=.88, Bartlett’s test of sphericity=1074.58, degrees of freedom=595, *p*<.001			

Scales	Items	Factor 1	Factor 2
		Negative atmosphere	Burden
Barriers	17. Difficulty speaking openly	.81	.35
	12. Doctors’ offices do not give information	.80	.39
	16. Difficulty obtaining information	.79	.41
	8. Distrust of health effects	.76	.19
	18. Negative reactions from people around you	.68	.46
	13. Attention-consuming	.33	.79
	5. High cost	.39	.76
	6. Time consuming	.45	.74
	4. Invisible effects	.30	.73
	3. Discomfort to reduce disposables	.23	.72
Eigenvalue		4.26	1.57
Total variance explained=58.5%			
Kaiser-Meyer-Olkin=.85, Bartlett’s test of sphericity=764.68, degrees of freedom=45, *p*<.001			

Scales	Items	Factor 1	Factor 2	Factor 3	Factor 4
		Lifestyle	Personal goods	Food	Dust
Personal health behavior	3. I reduced the use of chemicals.	.85	.43	.57	.33
	4. I avoid exposure to heavy metals.	.84	.49	.49	.29
	5. I use bisphenol-free products.	.81	.34	.49	.30
	1. I reduced antiseptic use.	.77	.36	.32	.29
	2. I avoid exposure to radiation.	.73	.28	.40	.35
	7. I keep away from cell phones.	.73	.49	.64	.27
	6. I avoid exposure to light at night.	.72	.35	.62	.20
	15. I avoid nail polish.	.42	.88	.45	.22
	13. I avoid air fresheners.	.34	.87	.34	.36
	14. I avoid antiseptic cosmetics.	.40	.81	.34	.34
	12. I avoid perfume.	.52	.67	.43	.19
	10. I reduce meat-eating.	.51	.34	.88	.31
	9. I eat a low-fat diet.	.45	.41	.87	.34
	11. I reduce food additives.	.55	.48	.73	.22
	18. I avoid micro-dust.	.30	.37	.30	.88
	17. I avoid car exhaust.	.38	.29	.39	.83
	16. I avoid cigarette smoke.	.33	.24	.20	.82
Eigenvalue		7.68	1.74	1.59	1.17
Total variance explained=67.7%					
Kaiser-Meyer-Olkin=.88, Bartlett’s test of sphericity=2154.69, degrees of freedom=153, *p*<.001					

Scales	Items	Factor 1	Factor 2	Factor 3	Factor 4
		Reduction	Involvement	Recycling	Reuse
Community health behavior	1. I reduce plastic use.	.89	.53	.35	.51
	2. I reduce waste.	.84	.42	.28	.34
	3. I reduce detergent use.	.82	.33	.38	.39
	4. I reduce disposables.	.81	.53	.34	.54
	5. I minimize personal use.	.78	.56	.40	.67
	16. I try to convince others to reduce disposables.	.53	.83	.25	.46
	13. I have an interest in environmental issues.	.49	.76	.17	.29
	17. I encourage others to separate garbage.	.35	.75	.44	.31
	18. I talk about ways to solve environmental problems.	.31	.74	.26	.37
	12. I participate in environmental activities.	.42	.73	.44	.50
	10. I separate waste collection.	.30	.30	.90	.35
	11. I separate battery waste.	.38	.26	.80	.42
	9. I separate drug waste.	.33	.43	.75	.21
	6. I reuse products.	.36	.36	.30	.88
	8. I conserve electricity.	.55	.46	.35	.87
	7. I conserve water.	.46	.55	.56	.62
Eigenvalue		6.89	1.56	1.45	1.09
Total variance explained=68.8%					
Kaiser-Meyer-Olkin=.87, Bartlett’s test of sphericity=1788.85, degrees of freedom=120, *p*<.001					

**Table 3.** Correlations among the eight women’s environmental health scales, Environmental Health Perception for Female Adolescents, and Environmental Health Behavior for Female Adolescents (N=210)

Scales	Scales, r (*p*)								
	1	2	3	4	5	6	7	8	9
2	.33	1							
	(<.001)								
3	.42	.56	1						
	(<.001)	(<.001)							
4	.11	.07	.28	1					
	(.110)	(.350)	(<.001)						
5	.06	.17	.17	.32	1				
	(.389)	(.016)	(.017)	(<.001)					
6	.16	.12	.13	.16	.17	1			
	(.018)	(.088)	(.069)	(.024)	(.017)				
7	.26	.40	.25	.44	.40	.10	1		
	(<.001)	(<.001)	(<.001)	(<.001)	(<.001)	(.151)			
8	.15	.06	.78	.66	.29	.21	.40	1	
	(<.001)	(.393)	(<.001)	(<.001)	(<.001)	(<.001)	(<.001)		
9	.62	.65	.78	.66	.29	.21	.40	.39	1
	(<.001)	(<.001)	(<.001)	(<.001)	(<.001)	(.002)	(<.001)	(<.001)	
10	.23	.55	.26	.51	.18	.10	.92	.90	.44
	(.001)	(<.001)	(<.001)	(<.001)	(.001)	(.013)	(<.001)	(<.001)	(<.001)

1=Severity; 2=susceptibility, 3=response efficacy; 4=self-efficacy; 5=benefit; 6=barrier; 7=personal behavior; 8=community behavior for women’s environmental health; 9=Environmental Health Perception for Female Adolescents; 10=Environmental Health Behavior for Female Adolescents.

## Supplementary material

Further details on supplementary materials are presented online (available at https://doi.org/10.4069/kjwhn.2021.06.21).

Supplementary Table 1. Item analysis of severity, susceptibility, response efficacy, self-efficacy, benefits, barriers, personal behavior, and community behavior for women’s environmental health (N=210)
