# Supplementary material for: Development and validation of women’s environmental health scales in Korea: severity, susceptibility, response efficacy, self-efficacy, benefit, barrier, personal health behavior, and community health behavior scales
Source: Korean J Women Health Nurs. 2021 Jun 30;27(2):153–65. doi: 10.4069/kjwhn.2021.06.21 (PMC9334188; doi:10.4069/kjwhn.2021.06.21)
Supplement: Supplementary Table 1. — Item analysis of severity, susceptibility, response efficacy, self-efficacy, benefits, barriers, personal behavior, and community behavior for women’s environmental health (N=210) [file kjwhn-2021-06-21-suppl.pdf]

Supplementary Table 1. Item analysis of severity, susceptibility, response efficacy, self-efficacy, benefits, barriers, personal behavior, and community behavior for women's environmental health (N=210)

| Scales | Subscales | Item no. | Mean | SD | Item-total γ | Factor-scale γ | Cronbach's α | Cronbach's α (95% CI) | Mean (SD) |
|---|---|---|---|---|---|---|---|---|---|
| Severity | Chemicals | 12 | 3.62 | 0.92 | 0.74 | 0.88 | 0.8 | 0.84 | 37.98 (5.66) |
| | | 9 | .3.39 | 0.96 | 0.72 | | | (.82–.88) | |
| | | 10 | 3.63 | 0.97 | 0.67 | | | | |
| | | 11 | 3.38 | 0.91 | 0.64 | | | | |
| | Electromagnetic waves | 8 | .4.00 | 0.79 | 0.71 | 0.79 | 0.74 | | |
| | | 7 | 3.74 | 0.82 | 0.68 | | | | |
| | | 23 | 4.54 | 0.61 | 0.62 | | | | |
| | Harmful food | 18 | 3.78 | 0.98 | 0.61 | 0.76 | 0.7 | | |
| | | 19 | 3.88 | 0.9 | 0.6 | | | | |
| | | 22 | 4.03 | 0.77 | 0.58 | | | | |
| Susceptibility | Reproductive health problems | 3 | 4.35 | 0.61 | 0.85 | 0.95 | 0.94 | 0.92 | 47.45 (5.56) |
| | | 2 | 4.36 | 0.63 | 0.82 | | | (.90–.94) | |
| | | 4 | 4.35 | 0.61 | 0.82 | | | | |
| | | 1 | 4.3 | 0.64 | 0.8 | | | | |
| | | 6 | 4.32 | 0.67 | 0.78 | | | | |
| | | 7 | 4.27 | 0.64 | 0.76 | | | | |
| | | 8 | 4.28 | 0.69 | 0.75 | | | | |
| | General health problems | 9 | 4.5 | 0.63 | 0.75 | 0.86 | 0.78 | | |
| | | 10 | 4.47 | 0.63 | 0.72 | | | | |
| | | 12 | 4.36 | 0.7 | 0.56 | | | | |
| | | 11 | 3.9 | 0.95 | 0.71 | | | | |
| Response efficacy | Avoid toxicant | 1 | 4.01 | 0.76 | 0.72 | 0.97 | 0.87 | 0.88 | 41.98 (5.50) |
| | | 12 | 3.89 | 0.93 | 0.74 | | | (.86–.91) | |
| | | 6 | 4.44 | 0.72 | 0.68 | | | | |
| | | 7 | 4.16 | 0.77 | 0.67 | | | | |
| | | 8 | 3.72 | 0.92 | 0.66 | | | | |
| | | 11 | 4.14 | 0.8 | 0.65 | | | | |
| | | 5 | 4.36 | 0.78 | 0.61 | | | | |
| | Pursuit of health | 2 | 4.48 | 0.67 | 0.72 | 0.86 | 0.76 | | |
| | | 3 | 4.32 | 0.73 | 0.73 | | | | |
| | | 4 | 4.45 | 0.7 | 0.5 | | | | |
| Self-efficacy | Preventive efficacy | 9 | 2.28 | 1.1 | 0.69 | 0.84 | 0.88 | 0.9 | 46.81 (8.81) |
| | | 6 | 3.17 | 0.93 | 0.67 | | | (.88–.92) | |
| | | 7 | 2.82 | 1 | 0.58 | | | | |
| | | 8 | 3.2 | 0.96 | 0.57 | | | | |
| | | 5 | 3.4 | 0.92 | 0.47 | | | | |
| | Judgement efficacy | 10 | 2.94 | 1.1 | 0.72 | 0.86 | 0.86 | | |
| | | 11 | 3.35 | 1.1 | 0.72 | | | | |
| | | 14 | 2.75 | 1.11 | 0.71 | | | | |
| | | 12 | 3.6 | 0.86 | 0.71 | | | | |
| | | 13 | 3.51 | 0.96 | 0.6 | | | | |
| | Control efficacy | 3 | 3.69 | 0.79 | 0.69 | 0.73 | 0.8 | | |
| | | 2 | 3.7 | 0.86 | 0.68 | | | | |
| | | 1 | 3.93 | 0.82 | 0.64 | | | | |
| | | 4 | 3.97 | 0.71 | 0.48 | | | | |
| Benefits | Psychological gainbenefits | 10 | 3.94 | 0.84 | 0.8 | 0.82 | 0.91 | 0.91 | 30.49 (5.35) |
| | | 9 | 3.95 | 0.84 | 0.79 | | | (.89–.93) | |
| | | 8 | 3.92 | 0.87 | 0.77 | | | | |
| | | 7 | 3.8 | 0.9 | 0.73 | | | | |
| | | 6 | 4 | 0.75 | 0.65 | | | | |

(Continued to the next page)

Supplementary Table 1. Continued

| Scales | Subscales | Item no. | Mean | SD | Item-total γ | Factor-scale γ | Cronbach's α | Cronbach's α (95% CI) | Mean (SD) |
|---|---|---|---|---|---|---|---|---|---|
| | Physical gainbenefits | 5 | 3.74 | 0.91 | 0.72 | 0.92 | 0.85 | | |
| | | 3 | 3.4 | 0.99 | 0.63 | | | | |
| | | 4 | 3.74 | 0.89 | 0.47 | | | | |
| Barriers | Negative atmosphere | 17 | 3.35 | 0.96 | 0.7 | 0.87 | 0.83 | 0.85 (.82–.88) | 33.42 (6.10) |
| | | 12 | 3.07 | 0.93 | 0.69 | | | | |
| | | 16 | 3.2 | 0.99 | 0.61 | | | | |
| | | 8 | 3.17 | 0.98 | 0.5 | | | | |
| | | 18 | 3 | 1 | 0.4 | | | | |
| | Burden | 13 | 3.49 | 0.89 | 0.68 | 0.84 | 0.81 | | |
| | | 5 | 3.49 | 0.95 | 0.67 | | | | |
| | | 6 | 3.54 | 0.89 | 0.64 | | | | |
| | | 4 | 3.71 | 0.87 | 0.6 | | | | |
| | | 3 | 3.4 | 1 | 0.57 | | | | |
| Personal health behavior | Lifestyle | 3 | 3.08 | 0.99 | 0.76 | 0.87 | 0.9 | 0.9 (.88–.92) | 55.11 (9.74) |
| | | 4 | 3.24 | 1.1 | 0.74 | | | | |
| | | 5 | 3.12 | 1.1 | 0.69 | | | | |
| | | 1 | 3.14 | 0.95 | 0.69 | | | | |
| | | 2 | 3.2 | 0.95 | 0.68 | | | | |
| | | 7 | 2.59 | 1.14 | 0.67 | | | | |
| | | 6 | 3.56 | 1.24 | 0.64 | | | | |
| | Personal goods | 15 | 3.23 | 1.15 | 0.63 | 0.77 | 0.84 | | |
| | | 13 | 3.16 | 1.03 | 0.65 | | | | |
| | | 14 | 3.14 | 1.22 | 0.64 | | | | |
| | | 12 | 2.96 | 1.12 | 0.59 | | | | |
| | Food | 10 | 2.7 | 1.09 | 0.67 | 0.82 | 0.82 | | |
| | | 9 | 3.32 | 1.03 | 0.66 | | | | |
| | | 11 | 3.79 | 0.96 | 0.65 | | | | |
| | Dust | 18 | 3.99 | 1 | 0.55 | 0.61 | 0.81 | | |
| | | 17 | 4.18 | 1.07 | 0.52 | | | | |
| | | 16 | 3.56 | 1.12 | 0.48 | | | | |
| Community health behavior | Reduction | 1 | 3.33 | 0.97 | 0.73 | 0.85 | 0.89 | 0.91 (.89–.93) | 55.10 (9.16) |
| | | 2 | 3.36 | 0.91 | 0.76 | | | | |
| | | 3 | 3.2 | 1 | 0.64 | | | | |
| | | 4 | 3.44 | 0.96 | 0.77 | | | | |
| | | 5 | 3.2 | 0.93 | 0.64 | | | | |
| | Involvement | 16 | 3.25 | 1.03 | 0.71 | 0.84 | 0.83 | | |
| | | 13 | 3.41 | 0.94 | 0.68 | | | | |
| | | 17 | 3.5 | 1.12 | 0.64 | | | | |
| | | 18 | 3.02 | 1.08 | 0.62 | | | | |
| | | 12 | 2.92 | 1.1 | 0.59 | | | | |
| | Recycling | 10 | 4.14 | 1.02 | 0.57 | 0.68 | 0.77 | | |
| | | 11 | 3.42 | 1.15 | 0.56 | | | | |
| | | 9 | 4.3 | 0.79 | 0.55 | | | | |
| | Reuse | 6 | 3.4 | 0.86 | 0.68 | 0.77 | 0.77 | | |
| | | 8 | 3.54 | 0.84 | 0.67 | | | | |
| | | 7 | 3.66 | 0.85 | 0.56 | | | | |

CI: Confidence interval.


https://doi.org/10.4069/kjwhn.2021.06.21
